# Pediatric Fracture Epidemiology in an Orthopaedic Urgent Care Setting

**DOI:** 10.1016/j.jposna.2025.100216

**Published:** 2025-05-31

**Authors:** Alexandra Abbott, Kate Swertfager, Summer Bloom, Nicholas J. Jackson, Joshua Goldman

**Affiliations:** 1 Division of Sports Medicine, University of California, Los Angeles, Los Angeles, CA, USA; 2 University of California, Los Angeles, Los Angeles, CA, USA; 3 Center for Sports Medicine, Orthopedic Institute for Children, Los Angeles, CA, USA; 4 Department of Medicine Statistics Core, University of California, Los Angeles, Los Angeles, CA, USA

**Keywords:** Fracture, Epidemiology, Pediatric, Musculoskeletal, Urgent care

## Abstract

**Background:**

Epidemiological data for pediatric fracture are limited, especially specific to the United States. Recent cohort studies from emergency departments and orthopaedic surgery clinics potentially skew data to more severe diagnoses. This retrospective cross-sectional study aims to update the epidemiology of pediatric fracture patients who presented to a pediatric orthopaedic urgent care center in an urban location.

**Methods:**

A total of 61,345 pediatric patients presented to an orthopaedics-focused urgent care from April 2019 to February 2023, and we analyzed data from 38,336 patients who received primary diagnoses of one or more fractures. Data were analyzed for associations between diagnoses and injury sites with age group, date of service, and sex.

**Results:**

Among more comprehensive epidemiological results, the mean age of patients with fractures was 9.7 years ± 4.3. Of the patients, 13,918 (36%) were female and 24,559 (64%) were male. School-aged children (aged 6 to 11 years) and adolescents (aged 12 to 18 years) each represented 40% (collectively 80%) of patients with fracture diagnoses. The most common fracture sites were of the wrist (27%), hand (18%), elbow (14%), and foot (12%). The most commonly injured bones were the radius (30%), hand phalanx (14%), and humerus (10%). Fracture subtype distribution included torus (13%), Salter-Harris (9%), avulsion (1%), and greenstick (less than 1%).

**Conclusion:**

This cohort study demonstrates disproportionate fracture presentation in males and in school-aged children (aged 6 to 12 years) and in adolescents (aged 12 to 18 years). We also demonstrate that radius and hand phalanx fractures are the most common in our pediatric cohort, together representing nearly half of fracture diagnoses.

**Key Concepts:**

(1) This study provides comprehensive and precise characterization of demographics and associations with pediatric fractures.
(2) This cohort, from a high-volume pediatric orthopaedic center, contributes robust and specific diagnostic data to improve upon prior studies’ characterization of epidemiology.
(3) Epidemiological characterization is important for guiding curriculums for clinician training, imaging pretest probabilities for various fracture diagnoses, and risk assessments based on demographics and injury sites.

**Level of Evidence:**

Level IV

## Introduction

Updated and specific epidemiological description for pediatric musculoskeletal injury is limited, especially in the United States and when examining fracture incidence. Limited recent studies often examine patient data from emergency, inpatient, and trauma center settings, which may limit generalizability to those with lower acuity [[1], [2], [3], [4], [5], [6], [7], [8], [9], [10], [11]]. These studies also share a common limitation of utilizing data from physicians not trained in orthopaedics or sports medicine; providers’ nonspecific diagnoses commonly describe body parts and nonspecific fracture types [1,3,7,11,12].

This retrospective cross-sectional study aims to update fracture epidemiology in the pediatric population. A volume of diverse patients treated at our institution presented with a wide spectrum of musculoskeletal injuries. Additionally, the urgent care, sports medicine clinics, and fracture clinics are staffed by providers with pediatric orthopaedic expertise. This allows for highly specific diagnoses and improved accuracy of available epidemiological data. The nature of our cohort allows for the potential improvement in accuracy and generalizability for pediatric populations.

## Materials and Methods

Initial visit data from the orthopaedic urgent care at the Luskin Orthopaedic Institute for Children in Los Angeles, California, were recorded from April 2019 to February 2023 for all patients who were diagnosed with fractures. This is a single-site urgent care staffed by physicians and by physician extenders (i.e., physician's assistants and nurse practitioners). This urgent care center is open Monday to Friday from 8 a.m. to 4 p.m. Patient ages in this cohort ranged from 0 to 18 years. Children were categorized as infant if 0 to 1 years old, toddler or preschool if 2 to 5 years old, school aged if 6 to 11 years old, and adolescent if 12 to 18 years old. Patient data were excluded if diagnoses were not specific enough to appropriately be categorized for epidemiological characterization, such as “pain,” “nonspecific injury,” and “unspecified joint.” Diagnoses were queried via International Classification of Diseases (ICD) codes associated with each visit. We also excluded patients who inappropriately presented to this center for non-musculoskeletal complaints and those older than 18 years to improve applicability to the pediatric population. A total of 69,216 patients' visit data for all musculoskeletal injury were recorded, and 38,477 fracture diagnosis visits were included for analysis.

Descriptive statistics (e.g., means with standard deviation and relative frequency) were used to analyze the cohort. Differences in demographics and fracture sites were examined between sex and age groups using t-tests (or analysis of variance) and chi-squares. Statistical significance was determined based on a two-sided α level of 0.05. All analyses were conducted in Stata version 17, Stata Corp, LLC (College Station, Texas).

## Results

### Fracture subtypes and sites

The most common fracture sites were of the wrist (27%), hand (18%), elbow (14%), and foot (12%). The most commonly injured bones were the radius (30%), hand phalanx (14%), and humerus (10%). Fracture subtypes analyzed included torus (13% of fractures), Salter-Harris (9%), avulsion (1%), and greenstick (less than 1%).

Data were also analyzed to provide fracture probability by site. Seventy-eight percent of upper extremity injuries and 45% of lower extremity injuries were diagnosed as fractures. For head injuries (limited to 7 cases in our nonemergency setting), 29% were fracture and 71% were nonfracture diagnoses. For the other sites, fracture diagnoses represented 5% of neck injuries, 8% of upper leg injuries, 17% of knee injuries, 26% of back injuries, 36% of upper arm injuries, 38% of ankle injuries, 41% of shoulder injuries, 66% of foot injuries, 72% of hand injuries, 73% of lower leg injuries, 79% of elbow injuries, 83% of forearm injuries, 87% of wrist injuries, and 98% of chest injuries (i.e., rib fractures).

### Age group differences

The mean age of our cohort of patients diagnosed with fracture was 9.7 years (SD: 4.3). Of the patients, 13,918 (36%) were female and 24,559 (64%) were male. School-aged children (aged 6–11 years) and adolescents (aged 12–18 years) each represented 40% (collectively 80%) of patients with fracture diagnoses. Infants (aged 0–1 years) represented 3% of fractures, and toddlers (aged 2–5 years) represented 17% of fractures. However, 78% of children aged 2 to 5 years presented with fracture, the highest proportion of age groups categorized in our cohort.

**Figure 1:**
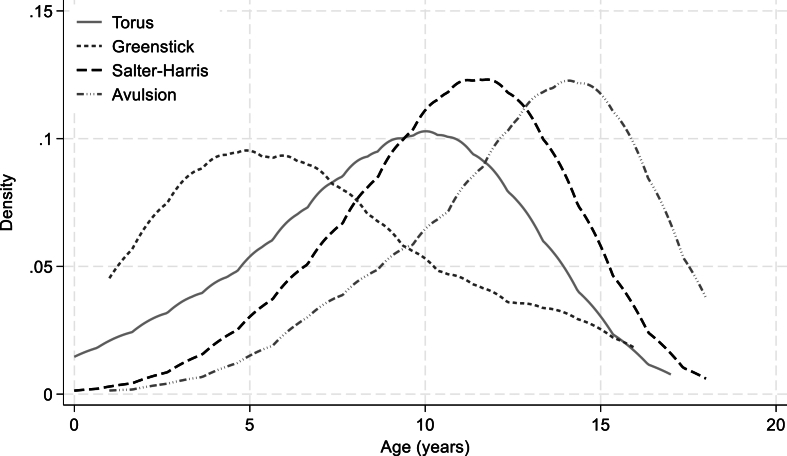
Fracture subtype age distribution.

Torus fractures were most common in infants (19.6% of infant fractures) and school-aged children (18.4% of fractures in school-aged children) (Fig. 1).

The mean age for torus fractures was 8.77 years (SD: 3.63). Torus fractures represented 8.2% of adolescent fractures and 11.4% of fractures in toddlers. Greenstick fractures represented 0.6% of infant fractures, 0.7% of toddlers' fractures, 0.3% of school-aged children's fractures, and 0.1% of adolescents’ fractures. The mean age for greenstick fractures was 6.91 years (SD: 4.00). Salter-Harris fractures represented 0.5% of infants’ fractures, 2.9% of toddlers' fractures, 11.3% of school-aged children's fractures, and 9.9% of adolescents’ fractures. The mean age for Salter-Harris fractures was 10.67 years (SD: 2.92). Avulsion fractures were most common in adolescents, representing 1.8% of fractures in this age group. The mean age for avulsion fractures was 12.67 years (SD: 3.17).

**Figure 2:**
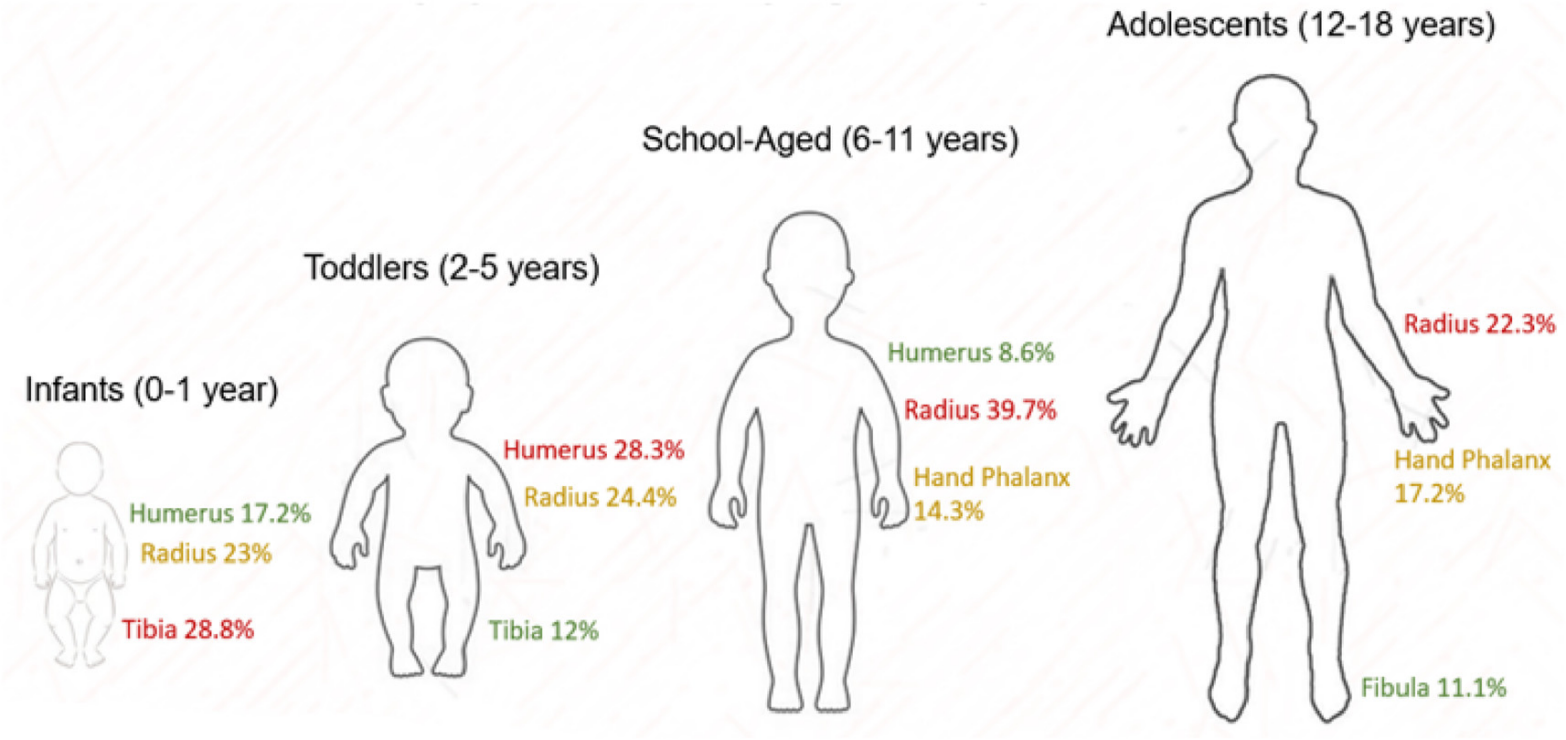
Fracture site distribution by age.

The most commonly fractured bones in infants were the tibia (28.8%), radius (23%), and humerus (17.2%) (Table 1). In toddlers, these were the humerus (28.3%), radius (24.4%), and tibia (12%). The most common fractures in school-aged children were of the radius (39.7%), hand phalanx (14.3%), and humerus (8.6%). Finally, the most common fractures in adolescents were of the radius (22.3%), hand phalanx (17.2%), and the fibula (11.1%, usually distal fibula/lateral malleolus) (Fig. 2). In all age groups, radius fractures were most commonly distal and humerus fractures were most commonly supracondylar.

**Table 1.** Most common fractures by age group.

	Age: 0-1 years	Age: 2-5 years	Age: 6-11 years	Age: 12-18 years
Calcaneus	0.0%	0.1%	0.2%	0.3%
Clavicle	9.9%	7.3%	3.4%	5.9%
Cuboid	0.2%	0.1%	0.2%	0.5%
Cuneiform	0.0%	0.1%	0.2%	0.1%
Femoral neck	0.0%	0.0%	0.0%	0.1%
Femur	2.4%	0.8%	0.3%	0.3%
Fibula	1.3%	3.2%	8.3%	11.1%
*Distal*	0.4%	1.6%	4.5%	4.4%
*Proximal*			0.0%	0.0%
Hamate	0.0%	0.0%	0.0%	0.1%
Hand phalanx	3.1%	5.8%	14.3%	17.2%
Humerus	17.2%	28.3%	8.6%	2.4%
*Proximal humerus*	0.8%	1.7%	1.8%	0.7%
*Shaft*	1.9%	0.2%	0.4%	0.5%
*Supracondylar*	10.9%	18.9%	4.1%	0.2%
*Lateral epicondyle*	2.0%	4.9%	0.8%	0.2%
*Medial epicondyle*		0.2%	0.5%	0.5%
*Transcondylar*		0.0%		0.0%
Pelvic ilium	0.0%	0.0%	0.0%	0.6%
Lunate	0.0%	0.0%	0.0%	0.1%
Metacarpal	0.8%	0.8%	1.9%	9.3%
Metatarsal	5.5%	7.4%	6.6%	7.0%
Foot navicular	0.0%	0.1%	0.3%	0.7%
Wrist navicular	0.0%	0.0%	1.9%	4.3%
Patella	0.0%	0.0%	0.3%	0.9%
Toe phalanx	1.5%	1.6%	4.0%	4.4%
Radius	23.0%	24.4%	39.7%	22.3%
*Proximal radius/radial head*	1.1%	5.4%	9.2%	7.1%
*Distal radius*	18.0%	16.0%	29.1%	14.4%
*Shaft*	3.9%	3.0%	1.4%	0.8%
Scapula	0.0%	0.0%	0.0%	0.2%
Talus	0.0%	0.2%	0.3%	1.1%
Tibia	28.8%	12.0%	5.4%	6.2%
*Distal*	6.2%	2.2%	3.5%	4.0%
*Proximal*	4.5%	3.8%	0.6%	1.6%
Tibia and fibula	0.0%	0.0%	0.1%	0.6%
*Trimalleolar*				0.1%
*Bimalleolar*			0.1%	0.5%
*Maisonneuve*				0.0%
Triquetrum	0.0%	0.0%	0.0%	0.1%
Ulna	6.2%	7.5%	3.4%	3.0%
*Proximal ulna*	1.6%	3.0%	1.0%	0.7%
*Shaft*	4.1%	4.0%	1.8%	0.9%
*Distal ulna*	0.6%	0.4%	0.7%	1.4%
Unspecified	0.2%	0.4%	0.5%	0.8%

### Sex differences

A total of 65.62% of boys who presented were diagnosed with fracture (24,418 fractures in 37,263 male patients). A total of 57.79% of girls who presented were diagnosed with fracture (13,918 fractures in 24,082 female patients). Thirty-six percent of patients seen for fracture were girls, and 64% were boys. The disparity increased with each age group (Table 2); there was nearly a 3:1 ratio of boys to girls in the adolescent age group with fractures (aged 12–18 years).

**Table 2.** Male to female fracture ratio by age groups.

	Percent male	Ratio of male to female
Age: 0-1 years	54.0%	1.17
Age: 2-5 years	55.4%	1.24
Age: 6-11 years	57.7%	1.36
Age: 12-18 years	74.4%	2.91

Boys were more likely to present with open fractures (1% of fractures and less than 1% for girls, *P* = .004). Girls were slightly more likely to present with torus fractures (14% of girls' fractures compared to 13% of boys’ fractures, *P* = .008) and Salter-Harris fractures (10% vs 9%, *P* = .002).

Site distribution was similar, with some exceptions. Boys were more likely to fracture the clavicle (6% of boys' fractures compared to 4% of girls’ fractures, *P* < .001), hand phalanx (14% vs 13%, *P* = .008), metacarpal (6% vs 2%, *P* < .001), and radius (31% vs 28%, *P* < .001).

Girls were more likely to fracture the fibula (10% of girls' fractures compared to 7% of boys’ fractures, *P* < .001), humerus (12% vs 8%, *P* < .001), wrist navicular (3% vs 2%, *P* = .001), toe phalanx (4% vs 3%, *P* < .001), and tibia (8% vs 7%, *P* < .001).

### Chronological data

**Figure 3:**
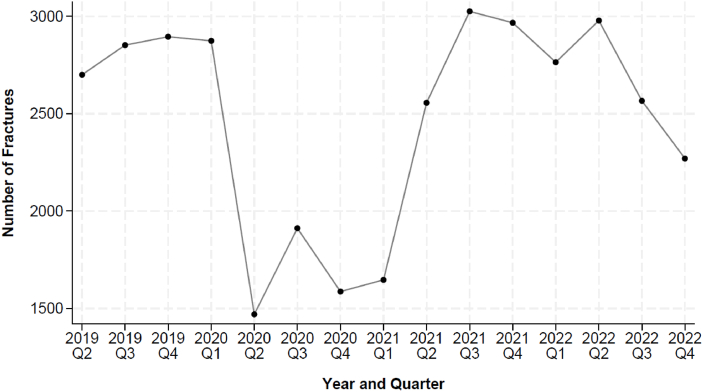
Fracture volume quarterly, 2019-2022.

Though not a primary aim of our study, maintaining four full-year data sets (2019-2022) through the COVID-19 pandemic demonstrated changes in encounter volume for fracture presentation by calendar year and by quarter (Fig. 3). Fracture counts from April-March 2019-2020, 2020-2021, 2021-2022, and 2022-2023 were 11,321, 6,616, 11,312, and 9.228, respectively (Table 3).

**Table 3.** Fracture volume annually, 2019-2023.

Year	Number of fracture patients
2019 (Apr 2019-Mar 2020)	11,321
2020 (Apr 2020-Mar 2021)	6,616
2021 (Apr 2021-Mar 2022)	11,312
2022 (Apr 2022-Feb 2023)	9,228

## Discussion

Pediatric fractures represent a substantial proportion of orthopaedic care, and understanding their distribution by age, sex, and injury site helps inform both clinical practice and public health efforts. This study offers a focused analysis of pediatric fractures seen at an orthopaedic urgent care center—a setting that differs from emergency departments and primary care settings in triage patterns, patient acuity, and diagnostic specificity. Our findings highlight important epidemiologic trends relevant to providers who manage pediatric fractures.

The mean age of our cohort was 9.7 years, and most fractures were in school-aged children and adolescents (aged 6 to 18 years). The peak age for girls was 5.3 years (81.2% fracture rate), and the peak age for boys was 4.3 years (79.7% fracture rate). Depending on the setting, peak and mean ages assessed in other studies are similar to our findings. In emergency department studies in the United States in 2010-2015, peak ages for fractures have been reported to be 6 to 12 years [1] and 10 to 14 years [12]. In an emergency department study in Switzerland of 1009 patients with fracture diagnoses, the mean age was 9 years and 7 months; peak ages were 9- to 12 years in girls and 12 to 14 years in boys [4]. Similarly, a recent large study of pediatric fractures in Israel demonstrated a peak age of 10- to 11 years in girls and 12 to 13 in boys [8]. The differences in peak ages of fracture incidences between boys and girls have been attributed to later onset of puberty in boys, with more rapid prepubertal growth likely coinciding with increased sport participation at different time points for adolescent boys and girls [8]. Two Chinese studies demonstrated relatively younger ages of peak incidence of 3- to 6 years [5] and of 6- to 12 years [7]. These studies involved fractures requiring hospital admission, which may be a factor for younger age. Both studies demonstrated “shoulder” and “distal humerus” to be the most common fracture in their cohorts, which may reflect the peak age for supracondylar humeral fracture (2-7 years) and its common need for admission [5,7].

Our overall fracture rate of 62% in our cohort is difficult to compare to limited recent studies [1,8,10,12,13]. Studies from 2010-2015 in emergency department settings demonstrate fracture rates of 11.5 to 15 per 1000 persons (1.2-1.5%) [1,12] and 1 in 5 children presenting to the emergency room diagnosed with a fracture (180 per 1000 children) [12]. A commonly cited statistic from studies of pediatric injuries in Canada and Finland in 2004 is that 10% to 25% of pediatric injuries are fractures [8,13]. Our fracture rate is considerably higher than 10% to 25%, likely related to patients presenting for only musculoskeletal injuries rather than injury in general. Our institution's strength in fracture care and availability of fracture clinics specifically is likely also contributory.

In our cohort, 36% of patients were girls and 64% were boys. Fifty-eight percent of girls who presented for musculoskeletal injury were diagnosed with fracture, compared to 66% of boys. The fracture risk in boys became increasingly discrepant with age: infants demonstrated a male-to-female ratio of 1.17 and adolescents demonstrated a ratio of 2.91. This is concordant with other studies, which demonstrate ratios up to 6:1 in adolescents, and similar or insignificant differences in infants [1,2,4,5,7,8,[10], [11], [12]]. The ratios demonstrated are higher in the previously mentioned Chinese studies of fractures requiring admission (5.3:1 and 5.7:1) [5,7]. Differences in fracture risk by sex may suggest pretest probabilities for providers but overall may have greater utility to inform public policy and injury reduction in sport.

The most common fractures for infants were of the tibia, radius, and humerus. Infants were also most likely to have torus fractures. Torus fracture incidence decreased in toddlers but increased again in school-aged children. A study in Israel utilizing visit data from multiple institutions in the country demonstrated that clavicle, skull, and femur fractures were the most common fractures in infants [8]. Similarly, specific studies of infant fractures have found the most common fractures to be of the skull, clavicle, and tibia/fibula [10,14]. Radius/ulna fractures and femur fractures both represented 5.9% of infant fractures in one infant study [14]. It is possible that these studies based on emergency and trauma center settings may represent clavicular and skull fractures more commonly than our urgent care setting. Our findings likely represent a lower acuity of infant fracture epidemiology. However, our results are concordant with these studies’ findings that infants commonly fracture long bones [8,14]. Eighty-two percent of fractures in the emergency department study resulted from falls from furniture [14]. Falling from furniture in infants and higher velocity falls in sport and play in school-aged children [15] may explain the bimodal peak of torus fractures we found in these discontinuous age groups.

In toddlers, the most common injuries were of the humerus, radius, and tibia, which is consistent with limited research globally [4,8,12]. Studies in the U.S. and in China examining pediatric fractures similarly noted that this age group most often requires admission for fracture management [1,5]. In an emergency department study in the U.S., an age younger than 6 years was significantly associated with the need for admission [1]. In a study of pediatric fractures in China, 42% of fractures requiring admission were in preschool-aged children [5]. As mentioned previously, this may reflect the common need for surgical intervention for supracondylar humeral fractures.

The most common injuries in school-aged children similarly included the radius and humerus, but hand phalanx injuries were more common in this older group than in toddlers. Studies of pediatric hand injuries in Japan [11] and Saudi Arabia [2] have similarly found hand fractures to be most common in school-aged and adolescent children. Most fractures in school-aged children have been found to have resulted from falls and from falls from heights, especially for supracondylar humeral fractures [15]. In our cohort, school-aged children were the most likely to present with physeal fractures. School-aged children experience increased injury forces and greater sports participation than toddlers, but are more likely to have open physes when compared to adolescents [15]. It has also been demonstrated that girls are more likely to sustain physeal fractures at a younger age than boys, corresponding to different pubertal onsets [15].

Adolescents in our study most commonly fractured the radius, hand phalanx, and fibula, and most commonly sustained avulsion fractures. These are the most common sites in other recent epidemiological studies including adolescents as well [8,12]. Avulsion fracture epidemiology is not well described, likely related to relatively rare incidence and to the specificity of diagnosis that is less common in general settings without orthopaedic emphasis.

We also observed a relative decrease in fracture care volume during 2020. This is consistent with national reports of decreased pediatric injuries during the COVID-19 pandemic, likely related to stay-at-home orders, school closures, and reduced participation in organized sports and recreational activities [3,11,12,16,17]. While our study was not designed to examine pandemic-related trends, the 2020 decrease in volume illustrates the extent to which injury patterns can shift in response to societal-level changes.

This study has important limitations to note and to inform future research goals. It reflects data from a single orthopaedic urgent care center and may not generalize to other care settings or geographic regions. Additionally, our dataset did not include mechanisms of injury or activity types, limiting our ability to assess contextual factors that may have contributed to specific injury patterns. Lastly, our institution's clinic triages clinically unstable fractures and those with a high likelihood for anesthesia needs to its adjacent facility for acute/emergency care. This likely results in under-representation of severe, open, and otherwise surgically indicated fractures in the population of interest.

Despite these limitations, our findings offer useful insights into contemporary patterns of pediatric fractures in an orthopaedic urgent care setting. Overall, fracture risk appears to be higher for boys, for school-aged and adolescent children, and for traumatic injuries involving the chest and upper extremities. Among the salient results discussed, we provide an update to pediatric fracture epidemiology in a cohort in the United States, with diagnostic specificity that contributes precise and comprehensive fracture data.

## Ethics approval and consent

The authors declare that no patient consent was necessary as no images or identifying information are included in the article.

## Author contributions

**Alexandra Abbott:** Writing – review & editing, Writing – original draft, Visualization, Supervision, Project administration, Methodology, Investigation, Formal analysis, Data curation, Conceptualization. **Kate Swertfager:** Writing – review & editing, Methodology, Investigation, Data curation. **Summer Bloom:** Writing – review & editing, Methodology, Investigation, Data curation. **Nicholas J. Jackson:** Writing – review & editing, Visualization, Validation, Software, Methodology, Formal analysis. **Joshua Goldman:** Writing – review & editing, Visualization, Supervision, Project administration, Methodology, Investigation, Data curation, Conceptualization.

## Funding

The research described was supported by NIH/National Center for Advancing Translational Science (NCATS) UCLA CTSI Grant Number UL1TR001881.

## Declaration of competing interests

The authors declare the following financial interests/personal relationships which may be considered as potential competing interests: Nicholas Jackson reports financial support was provided by National Center for Advancing Translational Sciences. If there are other authors, they declare that they have no known competing financial interests or personal relationships that could have appeared to influence the work reported in this paper.
